# Effects of Salinity and Eutrophication Variations on the Growth of *Myriophyllum spicatum*

**DOI:** 10.3390/plants14213305

**Published:** 2025-10-29

**Authors:** Meiqi Yin, Yipeng Wang, Huijia Song, Valeriia Dokuchaeva, Pan Wu, Lele Liu, Lele Lin, Weihua Guo

**Affiliations:** 1 Qingdao Key Laboratory of Ecological Protection and Restoration, Ministry of Natural Resources Key Laboratory of Ecological Prewarning, Protection and Restoration of Bohai Sea, School of Life Sciences, Shandong University, 72 Binhai Road, Qingdao 266237, China; 2 State Key Laboratory of Microbial Technology, Shandong University, Qingdao 266237, China; 3 Natural History Museum of China, 126 Tianqiao South Street, Beijing 100050, China; 4 Ecology and Nature Conservation Institute, Chinese Academy of Forestry, Beijing 100091, China

**Keywords:** eutrophication, salinity, large submerged plant, spiked watermilfoil, ecophysiological response, wetlands

## Abstract

Salinization and eutrophication are increasingly severe pollution problems in wetlands. *Myriophyllum spicatum* is a cosmopolitan species widely used for pollution control, but its physiological responses under combined stressors remain largely unknown. Here, we used mesocosms to investigate the ecophysiological responses of *M. spicatum* to three ammonia nitrogen concentrations (0, 1.5, and 3 mg L^−1^) and two salt concentrations (0 and 5 g L^−1^). Shoot and stem biomass were significantly affected by both salinity and nitrogen, whereas leaf phosphorus and stem nitrogen responded only to salinity (two-way ANOVA, *p* < 0.05). A significant salinity–nitrogen interaction was observed for stem biomass (*p* < 0.05); specifically, low nitrogen alone caused no significant reduction, but under saline conditions it markedly exacerbated biomass suppression. A significant salt–nitrogen interaction was detected for stem biomass (*p* < 0.05), such that low nitrogen alone did not significantly reduce stem biomass but exacerbated its suppression under saline conditions. These indicate potential synergistic environmental effects and suggest that even low nutrient inputs may aggravate stress under salt exposure. Stem biomass was significantly negatively correlated with malondialdehyde content (Pearson analysis, *p* < 0.05). Salt–nitrogen co-stress significantly increased malondialdehyde content (Tukey HSD test), indicating enhanced lipid peroxidation and associated oxidative damage, which may represent a physiological mechanism underlying growth inhibition in *M. spicatum*. Our findings demonstrate the complex adaptive responses of *M. spicatum* and emphasize the need to consider salt–nutrient interactions in conservation and restoration practices.

## 1. Introduction

Wetland ecosystems worldwide face escalating threats from the concurrent multiple pressures, particularly salinization and eutrophication [1]. Excessive nutrient inputs, mainly nitrogen (N) and phosphorus (P), from agricultural runoff and wastewater discharge have led to widespread eutrophication, resulting in harmful algal blooms [2] and mass mortality of hypoxia-sensitive species [3,4]. Simultaneously, irrigation practices, road de-icing salts, and seawater intrusion increase salinity concentrations in water systems, thereby disrupting osmotic balance and inducing ion toxicity in non-halophytic aquatic macrophytes [5,6]. These combined stressors trigger complex ecological responses that threaten aquatic biodiversity and ecosystem functioning like carbon sequestration [7,8,9]. Plant functional traits provide critical insights into physiological adaptation strategies under multiple stress conditions. Plants exhibit coordinated adjustments in key traits including growth parameter, photosynthetic parameters, antioxidant defenses, osmoregulatory compounds, and oxidative damage markers [10,11]. These traits frequently show integration through either synergistic enhancement or trade-off relationships, reflecting resource allocation priorities under stress constraints. Nitrogen is one of the most important nutrients influencing the growth and physiological performance of aquatic macrophytes. Among various nitrogen forms, ammonium (NH_4_^+^) can act as both a nutrient source and a potential stressor, depending on its concentration and the environmental context. At moderate concentrations, NH_4_^+^ serves as a readily available nitrogen source supporting photosynthesis and biomass accumulation; however, when accumulated at high levels or under alkaline conditions, the conversion of NH_4_^+^ to free ammonia (NH_3_) can induce physiological stress and oxidative damage in submerged macrophytes. In natural and constructed aquatic ecosystems, elevated salinity often co-occurs with increased nitrogen loading due to agricultural runoff, industrial discharges, or saline intrusion. The interaction between salinity and nitrogen can therefore produce synergistic or antagonistic effects on plant physiology, depending on the balance between osmotic adjustment and nitrogen metabolism. For instance, investment in defensive compounds is often traded off against growth-related traits, resulting in characteristic response patterns that elucidate underlying adaptive mechanisms. Globally, the extent of freshwater salinization and nutrient enrichment has been accelerating over the past decades, with reports showing that more than one-third of inland waters are already affected by salinity or nutrient pressures [2]. Such dual pressures not only compromise ecosystem services such as water purification and carbon storage, but also pose challenges to biodiversity conservation and wetland management at regional and global scales [4].

Eutrophication exerts both positive and negative influences on aquatic vegetation [12]. Excess ammonium (NH_4_^+^) has been demonstrated to be particularly toxic [13]. One of the major reasons is its effect on cellular pH homeostasis: the uptake and assimilation of ammonium release protons (H^+^) into the cytosol, leading to cytoplasmic acidification. This pH disturbance may reduce enzymatic activity and interfere with multiple metabolic pathways. By contrast, nitrate (NO_3_^−^) uptake is often coupled with proton consumption, which may alkalinize the cytoplasm. Excess ammonium disrupts cellular pH balance, inhibits photosystem II electron transport, and induces oxidative damage, ultimately reducing plant vitality and growth [14]. Notably, these negative effects intensify with exposure duration. Salinization induces multifaceted morphological and physiological disruptions in plants, including growth inhibition, decreased relative water content, reduced photosynthetic capacity, and altered stomatal behavior [15]. Concurrently, salt stress causes significant oxidative damage, manifested as elevated concentrations of malondialdehyde (MDA) and hydrogen peroxide (H_2_O_2_) [16]. MDA, a byproduct of lipid peroxidation, indicates oxidative damage to membrane lipids and thus reflects impairment of cellular membranes. In response, plants enhance antioxidant enzyme activities and accumulate osmolytes such as proline and flavonoids [17,18]. Furthermore, the accumulation of sodium (Na^+^) and chloride (Cl^−^) ions exacerbates ionic toxicity [16]. These changes ultimately affect key functional traits of wetland plants, thereby altering the structure and function of wetland ecosystems [19]. Although the primary mechanisms of these stressors differ, they converge on shared pathways of damage, most notably oxidative stress, yet manifest contrasting impacts on critical physiological functions [19]. Interactions between these stressors may result in additive, synergistic, or antagonistic effects on plant traits [19]. Notably, the extent to which osmotic adjustment compounds (e.g., soluble sugars, proline, and other compatible solutes) are integrated with oxidative stress markers (e.g., MDA) and antioxidant metabolites (e.g., flavonoids) under combined stress conditions remains insufficiently characterized. Although several recent studies have addressed multi-stressor effects on macrophytes, most investigations still emphasize biomass loss or single physiological responses. Comprehensive evaluations of trait integration under concurrent ammonium and salinity stress are still lacking, leaving an important gap in understanding the adaptive strategies of submerged plants. To date, most studies have concentrated on biomass responses rather than the coordination of functional traits, leaving a significant knowledge gap concerning how salt–ammonium interactions govern trait integration and, ultimately, plant performance.

*Myriophyllum spicatum*, a perennial submerged macrophyte of the Haloragaceae family, exhibits a cosmopolitan distribution across water systems, thriving in ponds, lakes, and rivers [7]. As a species that plays a fundamental role in N assimilation and water purification, *M. spicatum* represents an ideal model organism for investigating these interactions [14]. It demonstrates remarkable ecological adaptability, a high light saturation point, and significant nutrient removal capacity—effectively reducing concentrations of nitrogenous compounds (e.g., nitrite, nitrate, and ammonium) by up to 85% in contaminated waters [20,21]. Although the species possesses innate anatomical defenses against ammonium stress, such as well-developed aerenchyma facilitating internal oxygen transport, thicker cortical cell walls, and the capacity to sequester excess ions in central vacuoles, prolonged exposure intensifies its negative effects on population development [22]. In addition, while *M. spicatum* is recognized for its tolerance to moderate pollution, it undergoes pronounced morphological alterations under salinity stress, including apical meristem death and shoot dwarfing [23]. Nevertheless, its responses to combined salinity–ammonium stress remain poorly characterized, particularly with respect to the interactions between these stressors in shaping trait integration and adaptive strategies.

This study examines the multifactorial effects of NH_4_^+^-N and sodium chloride on *M. spicatum* through mesocosm experiments. We specifically test three hypotheses: (1) salt and ammonium are expected to inhibit the growth of *M. spicatum*, while simultaneously triggering adaptive physiological responses; (2) a significant interaction is expected between salt and ammonium stressors in shaping the physiology and growth of *M. spicatum*, rather than producing purely additive effects; and (3) the salt–ammonium interaction may modify the oxidative damage and osmoregulation, ultimately determining plant resilience. This study provides crucial insights for the management of aquatic vegetation in increasingly stressed freshwater ecosystems.

## 2. Results

### 2.1. Water Quality and Response of Plant Growth Traits

**Figure 1:**
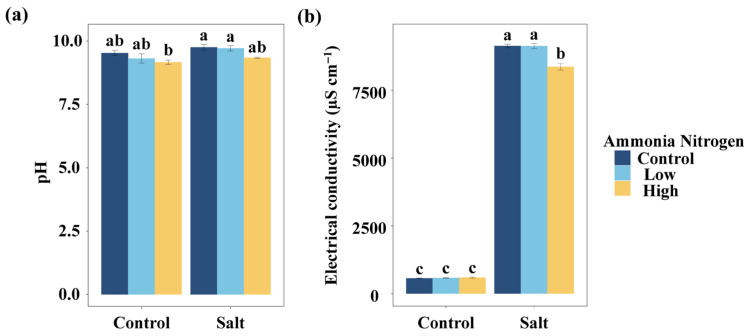
Comparisons of water pH (**a**) and electrical conductivity (**b**) among salt and ammonia nitrogen treatments. Different letters represent significant differences among treatments under multiple comparisons with the post-hoc Tukey HSD test (α = 0.05). Values are presented as Mean ± SE.

To evaluate how nitrogen contamination and salinity jointly influence basic water properties, we first examined pH and electrical conductivity, anticipating that both factors could alter water chemistry. Regardless of whether salt was added, the different concentrations of NH_4_^+^-N had no significant effect on the water pH (Figure 1a). Similarly, salt had no significant effect on the water pH at any NH_4_^+^-N concentration. Without salt, NH_4_^+^-N did not significantly affect water electrical conductivity (Figure 1b). Under saline conditions, the high NH_4_^+^-N concentration resulted in significantly lower electrical conductivity compared with the treatment without NH_4_^+^-N and with the low NH_4_^+^-N concentration. Salt addition significantly increased water electrical conductivity, regardless of the NH_4_^+^-N concentration (Figure 1b).

To assess plant growth responses, we analyzed morphological parameters and biomass allocation, expecting that both salt stress and nitrogen contamination would influence these traits either independently or interactively. Salt had significant effects on stem length, total biomass, shoot biomass, and stem biomass (Table 1). Shoot biomass, leaf biomass, and stem biomass were significantly influenced by NH_4_^+^-N (Table 1). In addition, the interaction of salt and NH_4_^+^-N significantly affected stem length and stem biomass (Table 1).

**Figure 2:**
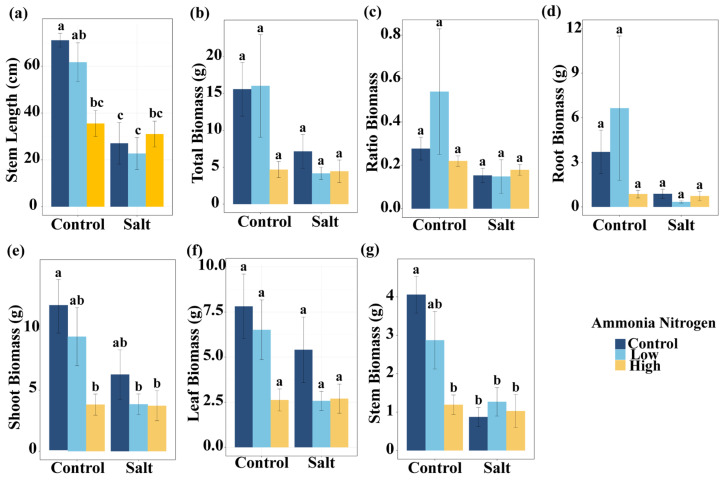
Comparisons of stem length (**a**), total biomass (**b**), ratio biomass (root–shoot biomass ratio) (**c**), root biomass (**d**), shoot biomass (**e**), leaf biomass (**f**), and stem biomass (**g**) among salt and ammonia nitrogen treatments. Different letters represent significant differences among treatments under multiple comparisons with the post-hoc Tukey HSD test (α = 0.05). Values are presented as Mean ± SE.

Under non-saline conditions, high NH_4_^+^-N concentration significantly reduced stem length compared with the treatment without NH_4_^+^-N. In the presence of salt, stem length did not differ among NH_4_^+^-N concentrations (Figure 2a). Salt markedly decreased stem length under the treatments without NH_4_^+^-N and with low NH_4_^+^-N concentration, but had no effect under high NH_4_^+^-N concentration (Figure 2a). Compared with the control (no salt, no NH_4_^+^-N), all treatments except low NH_4_^+^-N without salt significantly reduced stem length (Figure 2a). Under non-saline conditions, the high NH_4_^+^-N treatment significantly reduced shoot biomass compared with the treatment without NH_4_^+^-N. In the presence of salt, shoot biomass did not differ significantly among NH_4_^+^-N concentrations (Figure 2e). Relative to the control, shoot biomass was significantly reduced in the non-saline high NH_4_^+^-N treatment, as well as in the saline low- and high-NH_4_^+^-N treatments. For stem biomass, under non-saline conditions, high NH_4_^+^-N concentration resulted in a reduction compared with the treatment without NH_4_^+^-N (Figure 2e). Under salinity, stem biomass remained similar across NH_4_^+^-N concentrations. Salt decreased stem biomass in the absence of NH_4_^+^-N, but had no significant effect under low or high NH_4_^+^-N concentrations (Figure 2g). Compared with the control, low NH_4_^+^-N without salt showed no significant change, whereas the other treatments significantly reduced stem biomass (Figure 2g). Total biomass (Figure 2b), root-to-shoot ratio (Figure 2c), root biomass (Figure 2d), and leaf biomass (Figure 2f) did not differ significantly among salt and NH_4_^+^-N concentrations.

### 2.2. Response of Plant Physiological Traits

To explore how salinity and nitrogen contamination affect nutrient balance and stress physiology, we further examined nutrient contents as well as stress-related metabolites and enzyme activities. Salt had significant effects on the P content in leaves, leaf N:P, the N content in stems, and stem N:P (Table 2). Salt markedly influenced the activities or concentrations of several stress-related metabolites and enzymes, including the activity of superoxide dismutase (SOD), the content of MDA, the content of soluble sugars (SS), and the content of free amino acids (FAA) (Table 2). NH_4_^+^-N showed a significant effect on FAA (Table 2). The interaction of salt and NH_4_^+^-N significantly affected SS (Table 2).

**Figure 3:**
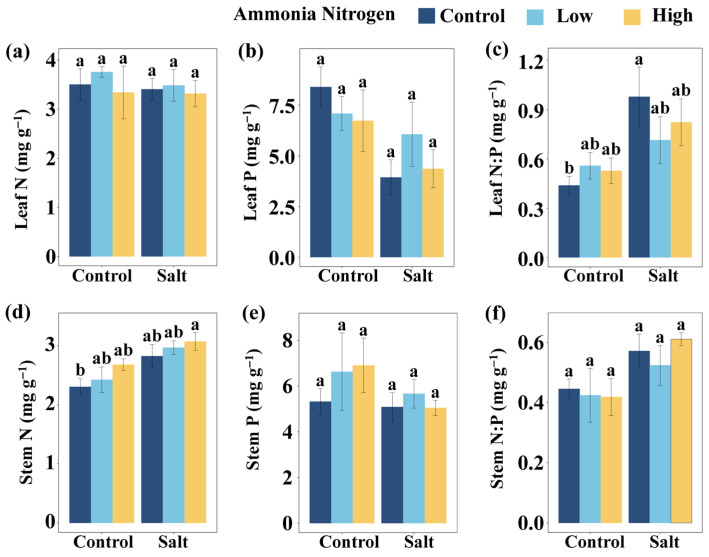
Comparisons of leaf N (**a**), leaf P (**b**), leaf N:P (**c**), stem N (**d**), stem P (**e**), and stem N:P (**f**) among salt and ammonia nitrogen treatments. N, nitrogen content; P, phosphorus content; N:P, ratio of N to P content. Different letters represent significant differences among treatments under multiple comparisons with the post-hoc Tukey HSD test (α = 0.05). Values are presented as Mean ± SE.

Leaf N:P did not differ significantly among NH_4_^+^-N concentrations under either saline or non-saline conditions (Figure 3c). In the absence of NH_4_^+^-N, salt addition markedly increased leaf N:P compared with the non-saline treatment, whereas under both low and high NH_4_^+^-N concentration no differences were observed between salt and non-salt conditions (Figure 3c). Relative to the control (no salt and no NH_4_^+^-N), a significant increase in leaf N:P occurred only when salt was applied without NH_4_^+^-N, while the other treatments all showed no significant effects (Figure 3c). Stem N content showed no significant variation among NH_4_^+^-N concentrations under either saline or non-saline conditions. Similarly, no differences were detected between salt and non-salt conditions at any NH_4_^+^-N concentration (Figure 3d). Compared with the control, stem N increased significantly only under the combination of salt and high NH_4_^+^-N concentration, whereas all other treatments had no significant effects (Figure 3d). Leaf N content (Figure 3a), leaf P content (Figure 3b), stem P content (Figure 3e), and stem N:P (Figure 3f) did not differ significantly among salt and NH_4_^+^-N treatments.

**Figure 4:**
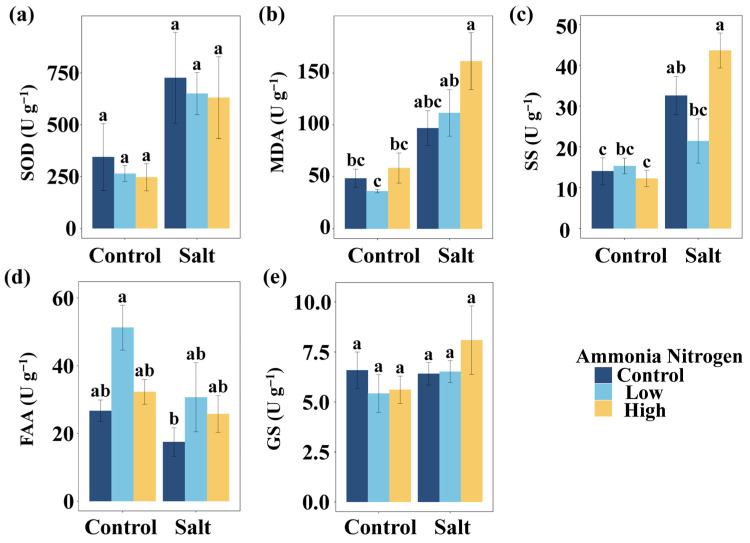
Comparisons of SOD (**a**), MDA (**b**), SS (**c**), FAA (**d**), and GS (**e**) among salt and ammonia nitrogen treatments. SOD, activity of superoxide dismutase; MDA, content of malondialdehyde; SS, content of soluble sugars; FAA, content of free amino acids; GS, activity glutamate synthetase. Different letters represent significant differences among treatments under multiple comparisons with the post-hoc Tukey HSD test (α = 0.05). Values are presented as Mean ± SE.

MDA content did not differ significantly among NH_4_^+^-N concentrations under either saline or non-saline conditions (Figure 4b). Under both low and high NH_4_^+^-N concentration, salt addition resulted in significantly higher MDA concentrations compared with the corresponding non-saline treatments (Figure 4b). Relative to the control (no salt and no NH_4_^+^-N), a significant increase in MDA occurred only under the combination of salt and high NH_4_^+^-N, while the other treatments had no effect (Figure 4b). SS content showed no significant differences among NH_4_^+^-N treatments under non-saline conditions (Figure 4c). Under salinity, SS content at high NH_4_^+^-N concentration was significantly higher than at low NH_4_^+^-N concentration (Figure 4c). Salt addition significantly increased SS concentrations in the absence of NH_4_^+^-N and under high NH_4_^+^-N concentration, whereas no difference was detected at low NH_4_^+^-N concentration (Figure 4c). Compared with the control, a significant increase in SS occurred only under salt without NH_4_^+^-N and salt with high NH_4_^+^-N, while the other treatments showed no significant effects (Figure 4c). SOD activity (Figure 3a), FAA content (Figure 3d), and glutamate synthetase (GS) activity (Figure 3e) did not differ significantly among the different NH_4_^+^-N concentrations under either saline or non-saline conditions, and their values were also not significantly different from the control.

### 2.3. Relationship Among Plant Traits

Results from the principal component analysis (PCA) showed that PC1 was primarily loaded by stem biomass (0.31), stem length (0.31), total biomass (0.30), shoot biomass (0.29), root biomass (0.27), and leaf biomass (0.27), while negative loadings were mainly contributed by stem N:P (−0.26), leaf N:P (−0.23), the content of MDA (−0.22), and the activity of SOD (−0.20). PC2 was characterized by positive loadings of leaf N (0.29), stem P (0.35), leaf P (0.28), and the content of FAA (0.21), whereas negative contributions were observed for leaf biomass (−0.31), the content of SS (−0.29), total biomass (−0.28), shoot biomass (−0.27), stem N:P (−0.27), and leaf N:P (−0.23). PC3 was mainly associated with positive loadings of the activity of glutamate synthetase (GS, 0.56), MDA (0.40), leaf P (0.27), and leaf N (0.20), in contrast to negative loadings of FAA (−0.29) and leaf N:P (−0.30) (Table A1).

**Figure A1:**
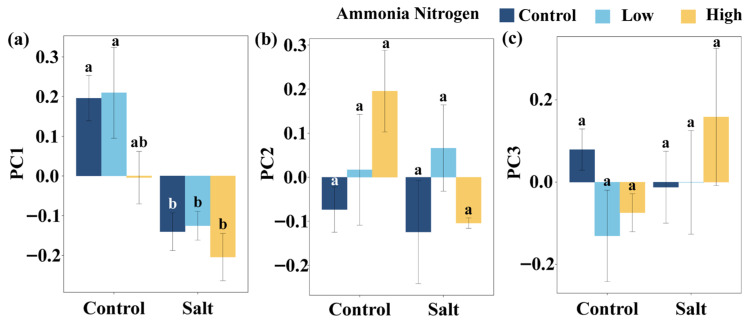
Comparisons of PC1 (**a**), PC2 (**b**), and PC3 (**c**) among salt and ammonia nitrogen treatments. Different letters represent significant differences among treatments under multiple comparisons with the post-hoc Tukey HSD test (α = 0.05). Values are presented as Mean ± SE.

Regardless of salinity, PC1 did not differ significantly among NH_4_^+^-N treatments. Under the treatments without NH_4_^+^-N and with low NH_4_^+^-N concentration, PC1 was significantly different between saline and non-saline conditions, whereas no such difference was detected under high NH_4_^+^-N supply (Figure A1a). PC2 (Figure A1b) and PC3 (Figure A1c) did not vary significantly among NH_4_^+^-N treatments under either saline or non-saline conditions.

**Figure 5:**
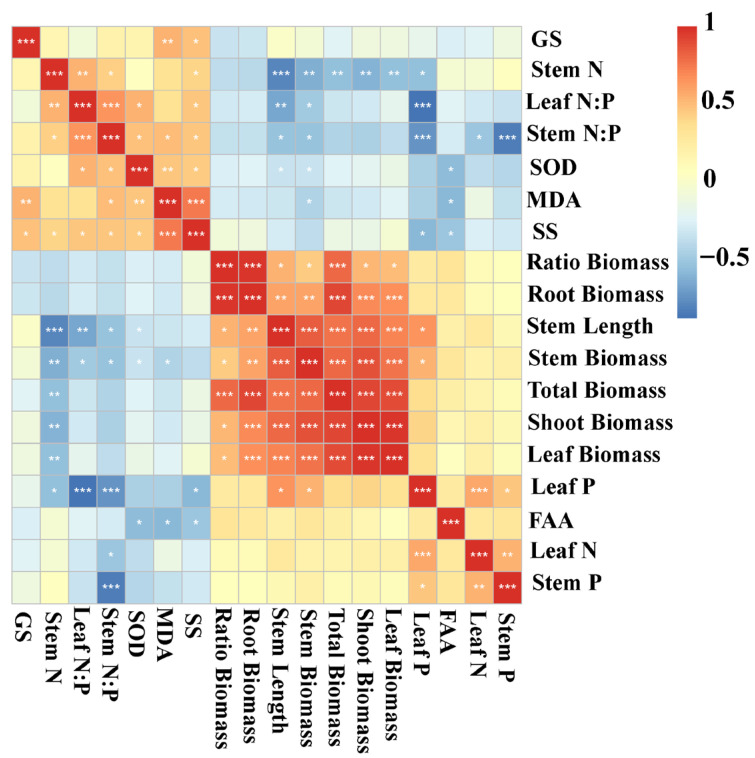
Heatmap for the autocorrelations of *Myriophyllum spicatum* traits. N, nitrogen content; P, phosphorus content; N:P, ratio of N to P content; SOD, activity of superoxide dismutase; MDA, content of malondialdehyde; SS, content of soluble sugars; FAA, content of free amino acids; GS, activity of glutamate synthetase; Ratio Biomass, root–shoot biomass ratio; Correlations are represented by shades of color with red being positive and blue being negative. The color closer to 1 or −1 represents a stronger correlation. * *p* < 0.05, ** *p* < 0.01, *** *p* < 0.001.

To further clarify the relationships among growth traits, nutrient status, and stress responses, correlation analyses were conducted to identify potential linkages between biomass allocation, nutrient content, and physiological indicators. Stem length was positively correlated with leaf P, leaf biomass, shoot biomass, stem biomass, root biomass, total biomass, and the root–shoot biomass ratio, and it was negatively correlated with SOD activity, stem N:P, leaf N:P, and stem N content. Stem biomass exhibited positive correlations with leaf P, leaf biomass, shoot biomass, root biomass, total biomass, and the ratio, but showed negative correlations with MDA, SOD activity, stem N:P, leaf N:P, and stem N content. Shoot biomass was positively correlated with leaf biomass, stem biomass, total biomass, root biomass, and the root–shoot biomass ratio, while being negatively correlated with stem N. Total biomass was positively associated with leaf biomass, shoot biomass, stem biomass, stem length, root biomass, and the root–shoot biomass ratio, but negatively associated with stem N content. Similarly, leaf biomass was positively correlated with shoot biomass, stem biomass, stem length, root biomass, total biomass, and the root–shoot biomass ratio, whereas it was negatively correlated with stem N content (Figure 5).

## 3. Discussion

NH_4_^+^-N has dual effects on submerged macrophytes: at low concentrations it functions as an essential nitrogen source [24], whereas at elevated levels it becomes phytotoxic, leading to the decline of submerged vegetation and the deterioration of freshwater ecosystems [4,25]. Our results support this dual role: low NH_4_^+^-N concentrations stimulated root biomass in *M. spicatum*, improving nutrient acquisition, whereas higher levels significantly suppressed shoot and stem biomass. Additionally, NH_4_^+^-N reduced pH, consistent with nitrogen-induced acidification commonly reported in terrestrial ecosystems. Under moderate NH_4_^+^-N stress, plants tended to allocate more biomass to roots to enhance nutrient uptake, a response that is consistent with the well-known stimulation of root growth under nitrogen deprivation. By contrast, under nutrient-rich conditions, plants usually reduce root allocation to mitigate environmental stress [24,26].

In contrast to previous studies suggesting that FAA mitigate ammonium toxicity [27,28], we found no significant differences in FAA concentrations among different NH_4_^+^-N treatments under non-saline conditions. Furthermore, other oxidative stress and osmotic adjustment indicators also showed no significant variation among NH_4_^+^-N treatments under non-saline conditions. These observations suggest that *M. spicatum* may exhibit metabolic adaptations when exposed solely to NH_4_^+^-N, with growth optimized through efficient N assimilation rather than relying on the more energy-consuming FAA accumulation mechanism. Such a strategy conserves carbon resources for biomass allocation to the roots, thereby enhancing nutrient foraging plasticity. Previous studies have reported marked increases in FAA concentrations under darkness (by 186%) [29] or excessive ammonium N [30], which may reflect energy-limited conditions where detoxification competes with photosynthesis for energy resources. In contrast, the lack of FAA accumulation observed in the present study may indicate that *M. spicatum* avoids additional metabolic costs by not activating further stress-response pathways, although this interpretation requires further experimental evidence to confirm. In the present study, the activity of SOD did not show significant changes under either salinity or NH_4_^+^-N treatments. This result suggests that *M. spicatum* may not primarily rely on SOD to mitigate oxidative stress under these conditions. Several explanations are possible: (i) the applied levels of stress may not have been sufficient to trigger an up-regulation of SOD; (ii) SOD activity could exhibit temporal fluctuations, and our sampling time may not have captured its peak response; or (iii) *M. spicatum* may preferentially activate alternative antioxidant mechanisms, such as CAT, POD, or non-enzymatic antioxidants (e.g., ascorbate, glutathione), to maintain redox balance. Previous studies have also reported stable or unchanged SOD activity in aquatic plants under specific stress conditions, indicating that the response of antioxidant enzymes is both species-specific and stress-dependent. Therefore, our findings highlight that the oxidative defense strategy of *M. spicatum* under the synergistic effects of salinity and nitrogen may rely mainly on enzymatic systems other than SOD.

Although the two-way ANOVA did not detect a statistically significant salt × N interaction (Table 2), post hoc multiple comparisons indicated that the combined high-salt and high-N treatment resulted in significantly higher MDA concentrations compared with the control (Figure 4). These findings suggest that although no global interaction was observed, the simultaneous presence of high salt and N may still intensify lipid peroxidation under certain extreme conditions. Salt stress significantly inhibited biomass accumulation and stem elongation in *M*. *spicatum*, consistent with previous findings [22,31]. The increase in MDA concentrations indicated that salt-induced oxidative stress may have led to membrane lipid peroxidation. Moreover, a significant negative correlation was observed between stem biomass and MDA content, suggesting that oxidative stress responses could be a potential mechanism limiting growth under stress conditions. The elevated SS content indicated a potential contribution to osmotic adjustment, which may represent one of the physiological strategies helping to maintain cellular homeostasis under salt stress. *Phragmites australis* is a widely distributed aquatic species, and previous studies have indicated that leaf water content can be a reliable predictor of its tolerance to salinity [32]. In the present study, we observed that lipid peroxidation in *M*. *spicatum* was significantly influenced by the interaction between salinity and nitrogen availability. These findings suggest that physiological indicators of stress tolerance in aquatic plants may exhibit species-specific patterns.

Under low concentrations of NH_4_^+^-N, the growth of *M. spicatum* was not significantly inhibited. However, when salt stress co-occurred, key growth traits such as shoot biomass, stem biomass, and stem length were markedly reduced. This indicates that although low concentrations of N can be absorbed and utilized to support growth, salinity may interfere with N use and ionic homeostasis, thereby exerting adverse effects on plant development. Previous studies have shown that elevated salinity disrupts ion balance and osmotic regulation and intensifies oxidative stress, ultimately constraining the growth and photosynthetic performance of submerged macrophytes [10,33,34]. Under such stress, plants often allocate resources between growth and defense, for instance by enhancing the synthesis of SS or antioxidants to improve osmotic adjustment and mitigate oxidative damage [35,36]. Therefore, nutrient control alone may not be sufficient to sustain the long-term recovery of dominant submerged species, and should be integrated with salinity regulation and broader watershed management. To our knowledge, this is the first study to experimentally demonstrate how the combined effects of low nitrogen concentration and salinity jointly influence the growth dynamics and physiological trade-offs of *M. spicatum*. This novelty provides new insight into the interactive mechanisms of nutrient and salt stress, and highlights the need to consider multiple environmental drivers simultaneously when designing management strategies for the restoration of submerged vegetation. Although the actual concentrations of ammonium and salinity differ across wetlands and seasons, these two stressors commonly co-occur due to agricultural inputs, wastewater inflow, seawater intrusion, or road-deicing activities. Therefore, controlling nutrient runoff—particularly limiting ammonium loading—could be a practical management strategy to alleviate multi-stressor effects on macrophyte growth and to support the restoration of submerged vegetation communities.

## 4. Materials and Methods

### 4.1. Experiment Design

**Figure 6:**
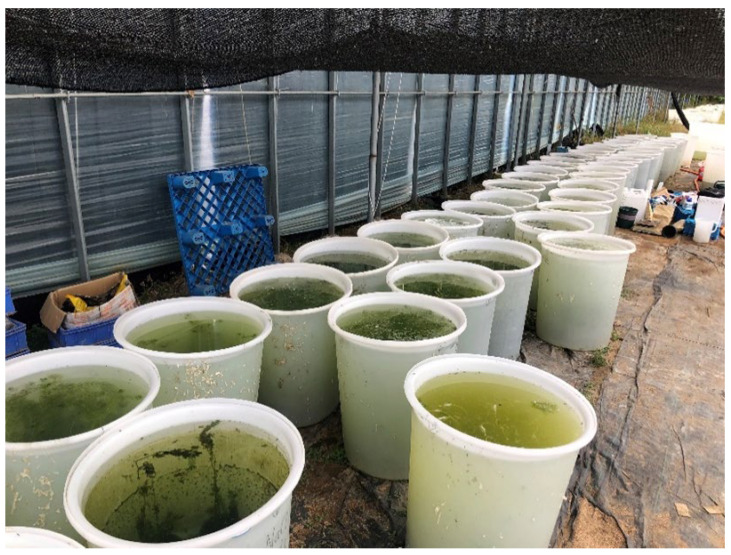
The internal view of the greenhouse with mesocosms.

The experiment was conducted in a greenhouse at Shandong University in Qingdao, Shandong Province, China (36°22′ N, 120°36′ E). The greenhouse covered approximately 666 m^2^ and was well ventilated. The average humidity and temperature in the greenhouse were approximately 80% and 28 °C, respectively. Specimens of *M*. *spicatum* were collected from Honghu Lake in Jingzhou, Hubei Province, China. To ensure uniformity, plants were cut into 25 cm segments, and six segments were planted in each 3.5 L pot at a depth of 5 cm. The substrate consisted of a 1:1 mixture of river sand and sediment, with 2.5 kg of substrate per pot. Pots were placed into 150 L water-filled buckets, with four pots per bucket (Figure 6).

The experiment was conducted from July to December 2023. After three months of growth, treatments were applied by adding ammonium chloride (NH_4_Cl) and sodium chloride (NaCl). To simulate nutrient conditions in wetlands, we established three N treatments: 0 mg L^−1^ as the control, 1.5 mg L^−1^ as the low-N treatment, and 3 mg L^−1^ as the high-N treatment. The concentrations were selected based on N concentrations observed between restored (≈3 mg L^−1^) and non-restored (≈6 mg L^−1^) zones of an urban wetland [37], while also considering that natural wetlands generally exhibit lower N concentrations. Considering the salinity range of 0.40–26.67 g L^−1^ in estuarine wetlands, we selected 5 g L^−1^ as the treatment concentration to simulate the typical habitat of *M. spicatum* while avoiding acute mortality [38,39]. We established two treatments: 0 g L^−1^ as the control and 5 g L^−1^ as the salt-addition treatment, which corresponds to salinity concentrations commonly observed in moderately saline soils of coastal and inland salt-affected regions. The culture medium was prepared using tap water that had been aerated for 48 h before use to remove residual chlorine and ensure dissolved oxygen equilibrium. No additional nutrients were supplemented except for the designed ammonium (NH_4_^+^) and salinity treatments. The full factorial design resulted in six treatment combinations. Each treatment combination was replicated five times, leading to a total of 30 buckets and 720 plants.

### 4.2. Parameter Measurements

In December, each pot was brought from the mesocosms. Plants were washed with tap water to remove sediment. Five healthy leaves from the upper-middle part of the tallest shoot in each pot were collected to determine the metabolites and antioxidant defense enzyme activity of *M. spicatum*. The contents of FAA and MDA were measured using a Solarbio assay kit (BC 5050 and BC 6410, respectively). The content of SS was measured by the anthrone colorimetry method [40]. The enzyme activities, including SOD (Solarbio, BC 5165) and glutamine synthetase activity (GS, Solarbio, BC 0915) were determined using assay kits.

The stem length of the five longest plants in each pot was measured using a ruler with an accuracy of 0.1 cm, and the average value was calculated. Subsequently, the plants were divided into stems, leaves, and roots, then oven-dried at 80 °C until a constant weight, and finally weighed to determine biomass.

The contents of N and P in the leaves and stems of *M. spicatum* were measured using a Kjeldahl N analyzer (K9860, Hanon, China) and the molybdenum blue colorimetric method (HJ 632-2011), respectively.

The electrical conductivity and pH of water was measured using a pH meter (FiveEasy Plus™, Mettler Toledo, Switzerland) and conductivity meter (FiveEasy Plus™, Mettler Toledo, Switzerland), respectively (ISO 10390: 2021 [41]; ISO 11265: 1994 [42]).

### 4.3. Statistical Analyses

All data analysis and visualization were conducted in R 4.2.3. Two-way ANOVA was employed to analyze the effects and interaction of salinization and eutrophication on plant traits. Differences in plant traits among the salt and ammonia N treatments were assessed using multiple comparisons, with Tukey’s HSD tests conducted via the R package *agricolae*. Before performing ANOVA and multiple comparisons, tests for normality and homogeneity of variances were conducted to ensure the validity of the statistical analyses. Principal component analysis (PCA) was used to examine trait differences across various gradients of salt and ammonia N treatments, performed using the R package *vegan*. Visualizations were completed with the R packages *ggplot2*. Each treatment included 20 pots, and the pot was considered as the experimental unit for statistical analysis.

## 5. Conclusions

This study demonstrates that salinity and ammonium interactively constrain the growth of *M. spicatum*, with combined stress intensifying oxidative damage and thereby limiting performance. These results highlight nutrient–salinity interactions as a critical factor shaping the resilience of submerged macrophytes. Recognizing such combined stressors will be essential for wetland management and restoration, where the persistence of aquatic vegetation under ongoing salinization and eutrophication is vital for sustaining ecosystem functions.

## Figures and Tables

**Table 1:** Two-way ANOVA for the growth traits of *Myriophyllum spicatum* under salt and ammonium nitrogen treatments.

	*F* (S)	*F* (N)	*F* (S × N)
Stem Length	28.871 ***	3.140	4.529 *
Total Biomass	6.336 *	2.293	1.348
Root–Shoot Biomass Ratio	3.282	0.802	1.085
Root Biomass	3.504	0.8620	1.092
Shoot Biomass	7.733 *	4.908 *	1.436
Leaf Biomass	4.054	4.277 *	0.989
Stem Biomass	21.348 ***	5.518 *	5.077 *

Notes: S, salt; N, ammonium nitrogen; S × N, the interaction of salt and ammonium nitrogen. *F* (S), *F* (N), and *F* (S × N) represent the *F* values from two-way ANOVA for the effects of salinity (S), nitrogen (N), and their interaction (S × N), respectively. * *p* < 0.05, *** *p* < 0.001.

**Table 2:** Two-way ANOVA for physiological traits of *Myriophyllum spicatum* under salt (S) and ammonium nitrogen (A) treatments.

	*F* (S)	*F* (N)	*F* (S × N)
Leaf N	0.178	0.373	0.074
Leaf P	6.392 *	0.399	1.047
Leaf N:P	11.178 **	0.197	1.410
Stem N	14.104 **	2.017	0.121
Stem P	1.336	0.680	0.341
Stem N:P	7.547 *	0.238	0.289
SOD	10.717 **	0.260	<0.001
MDA	27.188 ***	2.449	1.255
SS	26.417 ***	2.576	4.633 *
FAA	5.555 *	5.534 *	0.761
GS	1.753	0.421	1.078

Notes: S, salt; N, ammonium nitrogen; S × N, the interaction of salt and ammonium nitrogen; N, nitrogen content; P, phosphorus content; N:P, ratio of N to P content; SOD, activity of superoxide dismutase; MDA, content of malondialdehyde; SS, content of soluble sugars; FAA, content of free amino acids; GS, activity glutamate synthetase. *F* (S), *F* (N), and *F* (S × N) represent the *F* values from two-way ANOVA for the effects of salinity (S), nitrogen (N), and their interaction (S × N), respectively. * *p* < 0.05, ** *p* < 0.01, *** *p* < 0.001.

## Data Availability

Data will be made available upon request. The data are not publicly available due to privacy.

## Appendix A

**Table A1:** Principal component analysis of *Myriophyllum spicatum* traits.

	PC1	PC2	PC3
Stem N	−0.24	0.10	−0.10
Root–shoot Biomass Ratio	0.24	−0.14	−0.12
SS	−0.18	−0.29	0.30
Leaf N	0.14	0.29	0.20
Stem P	0.14	0.35	0.15
Root Biomass	0.27	−0.24	−0.14
FAA	0.15	0.21	−0.29
MDA	−0.22	−0.19	0.40
Leaf N:P	−0.23	−0.23	−0.30
Total Biomass	0.30	−0.28	−0.04
Leaf Biomass	0.27	−0.31	0.05
SOD	−0.20	−0.21	0.13
Shoot Biomass	0.29	−0.27	0.06
GS	−0.11	−0.08	0.56
Leaf P	0.24	0.28	0.27
Stem N:P	−0.26	−0.27	−0.18
Stem Biomass	0.31	−0.15	0.05
Stem Length	0.31	−0.14	0.17

Notes: N, nitrogen content; P, phosphorus content; N:P, ratio of N to P content; SOD, activity of superoxide dismutase; MDA, content of malondialdehyde; SS, content of soluble sugars; FAA, content of free amino acids; GS, activity of glutamate synthetase.
